# Association Between Left Atrial Volume Index (LAVI) and Glycemic Control (HbA1c) in Patients With Atrial Fibrillation and Type 2 Diabetes Mellitus

**DOI:** 10.7759/cureus.116179

**Published:** 2026-09-13

**Authors:** Marvelous O Popoola, Muhammad Abbas Shahid, Faiq Aftab, Muhammed Osikhena Oseni, Sheriff Adedolapo Solarin, Ayesha Ghazal Jamali, Doris Agbaragu, Eemaz Nathaniel, Basmala Moheieldeen Farouk Ismail, Abdul Hannan

**Affiliations:** 1 Accident and Emergency Medicine, Calvary Adelaide Hospital, Adelaide, AUS; 2 Cardiology, I.K. Akhunbaev Kyrgyz State Medical Academy, Bishkek, KAZ; 3 Cardiology, Glenfield Hospital University Hospitals of Leicester NHS Trust, Leicester, GBR; 4 Internal Medicine, Urgench State Medical Institute, Urgench, UZB; 5 Emergency Medicine, University of Ilorin College of Health Sciences, Ilorin, NGA; 6 Emergency Medicine, Dr. Soliman Fakeeh Hospital, Jeddah, SAU; 7 Emergency Medicine, King Fahad Specialist Hospital, Buraydah, SAU; 8 Medicine and Surgery, Liaquat University of Medical and Health Sciences, Jamshoro, PAK; 9 General Medicine, Imo State University, College of Medicine, Owerri, NGA; 10 General Medicine, Royal Blackburn Teaching Hospital, Blackburn, GBR; 11 Medicine, Watim Medical College, Rawalpindi, PAK; 12 Medicine, Cairo University Faculty of Medicine, Cairo, EGY; 13 Internal Medicine, University of South China, Hengyang, CHN; 14 Internal Medicine, Sheffield Teaching Hospitals NHS Foundation Trust, Sheffield, GBR

**Keywords:** atrial fibrillation, glycated hemoglobin, glycemic control, hba1c, left atrial remodeling, left atrial volume index, type 2 diabetes mellitus

## Abstract

Background: Glycemic control and left atrial remodeling in patients with concomitant type 2 diabetes mellitus (T2DM) and atrial fibrillation (AF) remain unclear. In this study, we examined the association between glycated hemoglobin (HbA1c) and left atrial volume index (LAVI) in this high-risk population.

Methods: A total of 700 patients with AF and T2DM were included in this retrospective study. We used medical records to obtain HbA1c and echocardiographic parameters. We assessed LAVI as a continuous variable and as a binary variable (enlarged LAVI > 34 mL/m²). We used Spearman correlation, multivariable linear regression, and binary logistic regression to examine the relationship between HbA1c and LAVI.

Results: There was a significant association between higher HbA1c and greater LAVI (Spearman's ρ = 0.287, p < 0.001). After multivariable adjustment, each 1% increase in HbA1c was associated with a 1.678 mL/m² increase in LAVI. Higher HbA1c was independently associated with increased odds of enlarged LAVI (adjusted odds ratio (OR) = 1.701, 95% confidence interval (CI): 1.391-2.081, p < 0.001). Hypertension, heart failure, and chronic kidney disease were also independently associated with enlarged LAVI.

Conclusion: In patients with concurrent AF and T2DM, higher HbA1c was independently associated with larger LAVI and higher odds of left atrial enlargement (LAE). Future prospective studies are warranted to determine whether improved glycemic control can influence atrial remodeling.

## Introduction

Atrial fibrillation (AF) is the most common chronic cardiac arrhythmia and is associated with a high risk of stroke, heart failure, and cardiovascular mortality [1]. Chronic hyperglycemia, inflammation, oxidative stress, and myocardial fibrosis are well-established pathways associated with increased AF risk, as they contribute to atrial structural and electrical remodeling in type 2 diabetes mellitus (T2DM) [2]. These remodeling processes create a pro-arrhythmic substrate that facilitates AF induction, maintenance, and perpetuation [3,4].

Left atrial volume index (LAVI) is a sensitive echocardiographic marker of left atrial (LA) remodeling and is superior to linear LA dimensions associated with adverse cardiovascular outcomes [5,6]. The association between glycated hemoglobin (HbA1c), a standard glycemic control marker, and cardiovascular remodeling has been reported but remains poorly explored in patients with both AF and T2DM [7,8].

HbA1c is a glycated form of hemoglobin formed through the non-enzymatic attachment of glucose to hemoglobin. Because hemoglobin within erythrocytes has an average circulating lifespan of approximately 120 days, HbA1c generally reflects average glucose levels over the preceding 60-90 days. It is widely used to assess long-term glycemic control [9]. Higher HbA1c levels have been associated with microvascular and macrovascular complications in patients with diabetes. Persistent hyperglycemia may promote oxidative stress, inflammation, and endothelial dysfunction, which may contribute to cardiovascular injury and remodeling. These metabolic disturbances may also contribute to myocardial and atrial structural remodeling [10,11].

Recent studies have indicated that poor glycemic control could also lead to adverse cardiovascular outcomes in people with diabetes and might accelerate atrial remodeling [8,12]. HbA1c is associated with atrial fibrosis and LA enlargement, reflecting atrial structural remodeling and potentially contributing to the development and progression of AF [8,13]. Studies have reported a relationship between HbA1c and atrial structural remodeling and LA enlargement in patients with T2DM. However, limited research has specifically examined the link between these two parameters, LAVI and HbA1c, in people with both AF and T2DM [13,14]. Thus, the association needs further exploration to better understand its clinical relevance.

This study investigates the relationship between HbA1c and LAVI, especially among patients with both AF and T2DM, which has not been studied extensively. The findings could help determine whether poor glycemic control is significantly associated with LA remodeling and improve cardiovascular risk stratification. This study explored the association between HbA1c and LAVI in patients with AF and T2DM.

## Materials and methods

Study design and setting

The study design was a hospital-based observational cross-sectional design with retrospective data collection from patients' medical records. The study was conducted at Watim Medical and Dental Hospital, a tertiary care hospital, in the Department of Cardiology, where patients admitted with AF and T2DM routinely underwent detailed clinical assessment, laboratory investigations, and transthoracic echocardiography (TTE). The study evaluated how well HbA1c levels relate to LAVI, a known measure of LA structural remodeling, using hospital medical records. Data collection followed the necessary institutional approvals and included a review of electronic and paper medical records of eligible patients.

Study population

The study population included adults with a known history of AF and T2DM who were followed in the cardiology department. The study was conducted between January 2026 and July 2026. We included patients with complete clinical, laboratory, and echocardiographic records. The final analysis included 700 patients who met the eligibility criteria.

Sample size and sampling technique

We identified eligible medical records using a consecutive sampling approach during the predefined study period. During the study period, we screened 1,000 medical records. Of these, 80 did not have HbA1c data, 70 did not have available LAVI measurements, 60 had missing clinical data, and 90 were excluded due to predefined structural heart disease (SHD) or other exclusion criteria. Lastly, 700 patients were analyzed. Figure 1 summarizes the selection of study records and reasons for exclusion. No a priori sample size calculation was performed, as this was a retrospective study of all eligible records available over the predetermined study period. Selection bias was possible due to incomplete records, as only records with complete information were analyzed.

**Figure 1:**
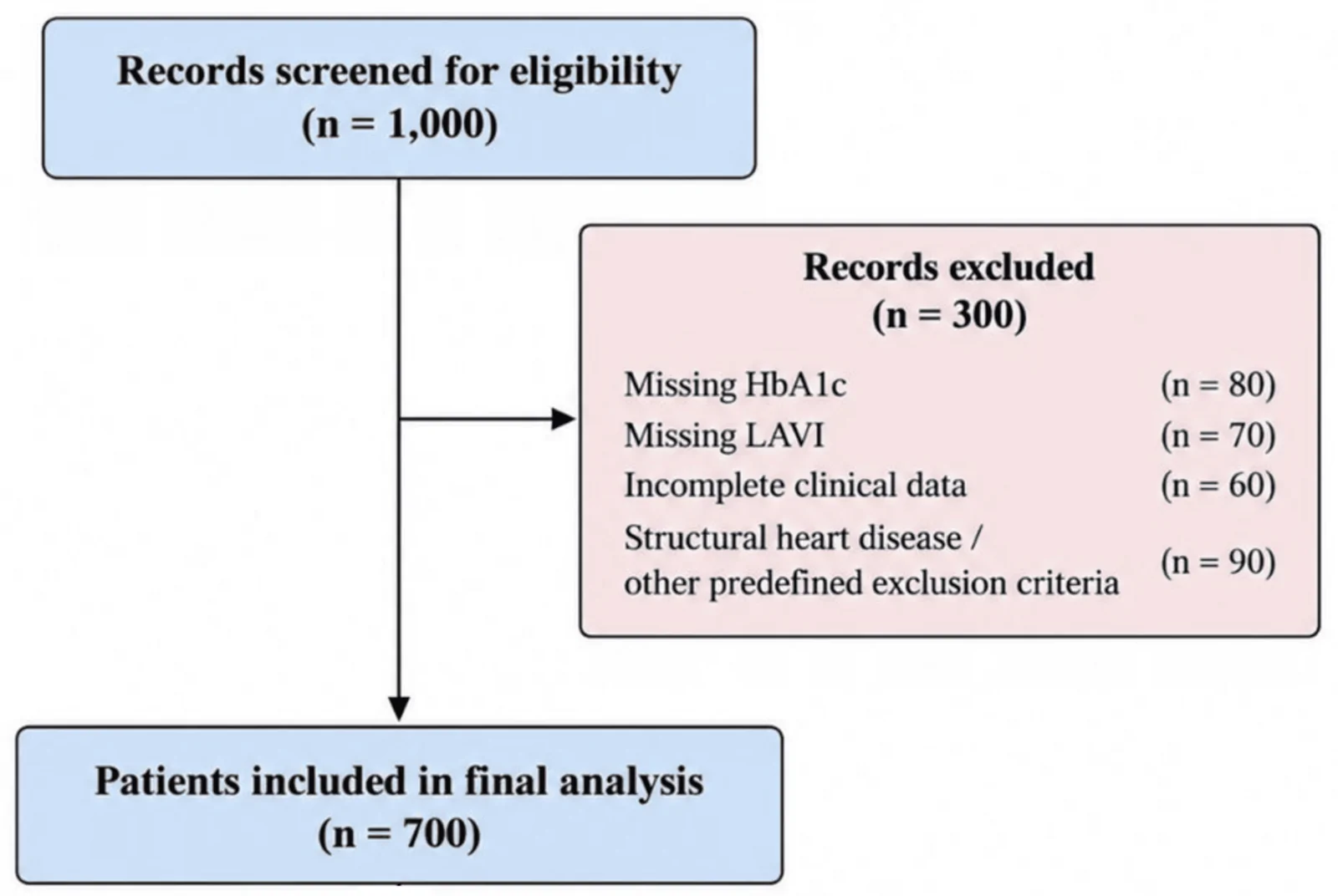
Flow diagram of patient selection and exclusion. LAVI: left atrial volume index

Eligibility criteria

The patients comprised adults aged 18 years or older with a confirmed diagnosis of AF and T2DM. We considered AF confirmed when documented in the medical record as a physician diagnosis supported by electrocardiographic or rhythm-monitoring evidence. We considered T2DM confirmed when documented in the medical record according to established clinical diagnostic criteria or a previous physician diagnosis. Patients had to have a documented HbA1c as part of routine clinical care and a comprehensive TTE examination, including measurement of the LAVI. We included records with complete demographic, clinical, laboratory, and echocardiographic data.

We excluded patients without complete or available medical records, those without HbA1c, and those without available LAVI. Patients with other SHD that predisposes to AF, such as congenital heart disease or previous cardiac surgery that affects LA anatomy, were also excluded, as were patients with significant other valvular heart disease requiring intervention, and those with other conditions that could substantially alter LA dimensions independent of diabetes.

Data collection procedure

After Institutional Review Board approval, we identified patient records in the hospital medical record system. We collected demographic characteristics, clinical history, laboratory parameters, and echocardiographic parameters on a standardized data collection form designed for this study. HbA1c values were obtained from routine laboratory reports recorded in the medical records. HbA1c was reported as a percentage (%). We analyzed it as a continuous variable in the regression analyses. In contrast, we extracted echocardiographic variables, including LA volume and LAVI, from TTE reports prepared by experienced cardiologists. Experienced cardiologists obtained echocardiographic measurements from routine clinical echocardiography reports. Because this retrospective study used previously recorded echocardiographic measurements, we did not perform a formal intraobserver or interobserver reproducibility assessment. We used the biplane disk method (Simpson's method) to measure LA volume from the apical four-chamber and two-chamber views, then calculated LAVI based on body surface area. Because all participants had AF, we obtained echocardiographic measurements by averaging five consecutive cardiac cycles to account for beat-to-beat variability. Measurements were taken according to the echocardiographic protocol used at the study institution. We cross-checked all extracted data for completeness and accuracy before entering them into the study database. We removed patient identifiers before data analysis to maintain confidentiality. For patients with more than one eligible HbA1c or echocardiographic measurement during the study period, we used the measurement corresponding to the same clinical assessment episode for analysis. Each patient contributed only one observation to the final dataset.

Data quality control

All extracted information was independently reviewed for accuracy and consistency before being entered into the database. All data were verified for missing, duplicate, and inconsistent data. In cases of discrepancy, we re-examined the original medical records; the final analysis used full datasets that met all study criteria.

Statistical analysis

We used IBM SPSS Statistics version 26 (IBM Corp., Armonk, NY, USA) for data analysis. Data were presented as mean ± standard deviation (SD) or median and interquartile range (IQR) for continuous variables, and as frequencies and percentages for categorical variables. We used Spearman's rank correlation to assess the association between HbA1c and LAVI. We used multiple linear regression to assess the independent association between HbA1c and LAVI after adjusting for age, body mass index (BMI), duration of diabetes, hypertension, coronary artery disease (CAD), heart failure, chronic kidney disease, left ventricular ejection fraction (LVEF), AF type, and diabetes treatment. We excluded diastolic dysfunction from the primary model because its assessment may incorporate LAVI-related parameters and could introduce circularity or overadjustment. We then performed a sensitivity analysis by additionally adjusting for diastolic dysfunction. Before fitting the multivariable linear regression model, we checked assumptions of linearity, normality, and homoscedasticity of residuals, independence of observations, and the absence of problematic multicollinearity using appropriate diagnostic procedures. We identified no major violations of linear regression assumptions, and variance inflation factors (VIF) did not indicate problematic multicollinearity. We compared LAVI and glycemic control groups using the Mann-Whitney U test and used binary logistic regression to identify factors associated with enlarged LAVI. We selected demographic, metabolic, cardiovascular, renal, and echocardiographic parameters as covariates a priori because of their known clinical relevance to glycemic status and LA remodeling. We entered HbA1c as a continuous variable (percentage points) in the multivariable linear and logistic regression models. We analyzed LAVI as a continuous variable (mL/m²) in the linear regression model and as a binary outcome (≤34 vs. >34 mL/m²) in the logistic regression model. The final logistic regression model included 13 predictor parameters and 465 participants with enlarged LAVI, corresponding to approximately 35.8 events per parameter. A two-tailed p-value of <0.05 was considered statistically significant.

Ethical considerations

Before the study, the Institutional Review Board of Watim Medical College approved the study (WMC-IRB-0091-2026). Because this investigation used retrospectively collected, de-identified medical records, there was no direct interaction with patients or study-related intervention. The study maintained patient confidentiality and privacy by removing all patient information before data extraction and analysis. Only the research team had access to the study data, and all procedures were carried out in accordance with the ethical principles of the Declaration of Helsinki and the research ethics guidelines of the institution where the study involving human participants was conducted.

Operational definitions

Glycated hemoglobin (HbA1c) was used as a marker of glycemic control and presented as a percentage, representing the average blood glucose over the last 2-3 months. LA remodeling was assessed using LAVI, expressed in mL/m² and calculated by indexing LA volume measured using the biplane Simpson’s method to body surface area. Higher LAVI values indicate greater LA volume and remodeling. We classified diastolic function as normal, Grade I, Grade II, or Grade III based on echocardiographic assessments documented in the patients’ medical records [15]. In categorical analysis, LAVI ≤ 34 mL/m² was assigned to the normal group, and LAVI > 34 mL/m² was assigned to the enlarged group, based on the established echocardiographic recommendations [15].

## Results

Table 1 summarizes the descriptive statistics of the continuous study variables. The mean age of participants was 64.16 ± 8.72 years, with a median of 64.00 years (IQR, 12.00). The mean BMI was 27.95 ± 3.93 kg/m², while the mean duration of diabetes was 9.34 ± 5.74 years. The mean HbA1c was 7.88% ± 1.19%. Regarding echocardiographic parameters, the mean LA volume was 70.89 ± 18.34 mL, mean LAVI was 37.85 ± 8.47 mL/m², and mean LVEF was 57.50% ± 6.01%. Overall, the values demonstrated moderate variability across the continuous study variables.

**Table 1:** Descriptive statistics for the continuous study variables (N = 700). M: mean; SD: standard deviation; CI: confidence interval; IQR: interquartile range; BMI: body mass index; HbA1c: glycated hemoglobin; LAVI: left atrial volume index; LVEF: left ventricular ejection fraction

Variable	M ± SD	95% CI for mean	Median	IQR	Range
Age (years)	64.16 ± 8.72	(63.51, 64.80)	64.00	12.00	40-86
BMI (kg/m²)	27.95 ± 3.93	(27.66, 28.25)	28.00	5.20	18.50-39.40
Duration of diabetes (years)	9.34 ± 5.74	(8.91, 9.76)	9.05	8.00	1-30
HbA1c (%)	7.88 ± 1.19	(7.79, 7.96)	7.90	1.70	5.40-11.50
Left atrial volume (mL)	70.89 ± 18.34	(69.52, 72.25)	69.80	24.63	30.80-138.70
LAVI (mL/m²)	37.85 ± 8.47	(37.22, 38.48)	37.60	11.20	19.00-66.70
LVEF (%)	57.50 ± 6.01	(57.06, 57.95)	57.90	7.90	35.20-75.00

Table 2 presents the categorical characteristics of the study participants. Of the 700 participants, 370 (52.9%) were male, and 330 (47.1%) were female. Regarding diabetes treatment, 294 (42.0%) were taking oral medications, 151 (21.6%) were taking insulin, and 255 (36.4%) were taking both. Paroxysmal, persistent, and permanent AF were present in 190 (27.1%), 259 (37.0%), and 251 (35.9%) participants, respectively. Diastolic dysfunction was absent in 276 (39.4%) participants, whereas Grade I, II, and III dysfunction was observed in 174 (24.9%), 156 (22.3%), and 94 (13.4%), respectively. Hypertension was present in 346 (49.4%), CAD in 198 (28.3%), heart failure in 130 (18.6%), and chronic kidney disease in 113 (16.1%) participants. One hundred seventy-nine (25.6%) participants reported smoking. A total of 543 (77.6%) participants had poor glycemic control (HbA1c ≥ 7%). Based on the predefined LAVI threshold, 465 (66.4%) participants had an enlarged LAVI (>34 mL/m²), while 235 (33.6%) had a normal LAVI (≤34 mL/m²).

**Table 2:** Categorical characteristics of the study participants (N = 700). Percentages are based on the total sample of 700 participants. There were no missing values for the variables shown. HbA1c: glycated hemoglobin; LAVI: left atrial volume index

Variable	N	%
Sex		
Male	370	52.9
Female	330	47.1
Diabetes treatment		
Oral drugs	294	42.0
Insulin	151	21.6
Both oral drugs and insulin	255	36.4
Atrial fibrillation type		
Paroxysmal	190	27.1
Persistent	259	37.0
Permanent	251	35.9
Diastolic dysfunction grade		
Normal	276	39.4
Grade I	174	24.9
Grade II	156	22.3
Grade III	94	13.4
Hypertension		
No	354	50.6
Yes	346	49.4
Coronary artery disease		
No	502	71.7
Yes	198	28.3
Heart failure		
No	570	81.4
Yes	130	18.6
Chronic kidney disease		
No	587	83.9
Yes	113	16.1
Smoking		
No	521	74.4
Yes	179	25.6
Glycemic control category		
Good control, HbA1c < 7%	157	22.4
Poor control, HbA1c ≥ 7%	543	77.6
LAVI category		
Normal, LAVI ≤ 34 mL/m²	235	33.6
Enlarged, LAVI > 34 mL/m²	465	66.4

Figure 2 shows a positive link between HbA1c and LAVI, with substantial variability in LAVI across HbA1c values. There is considerable variation in individual observations around the regression line. Still, the general upward trend agrees with the statistically significant positive correlation found in the correlation analysis (Spearman's ρ = 0.287, p < 0.001). This implies that poorer glycemic control is also linked with increased LAVI, but the variability of the data suggests other clinical and demographic variables also influence LAVI.

**Figure 2:**
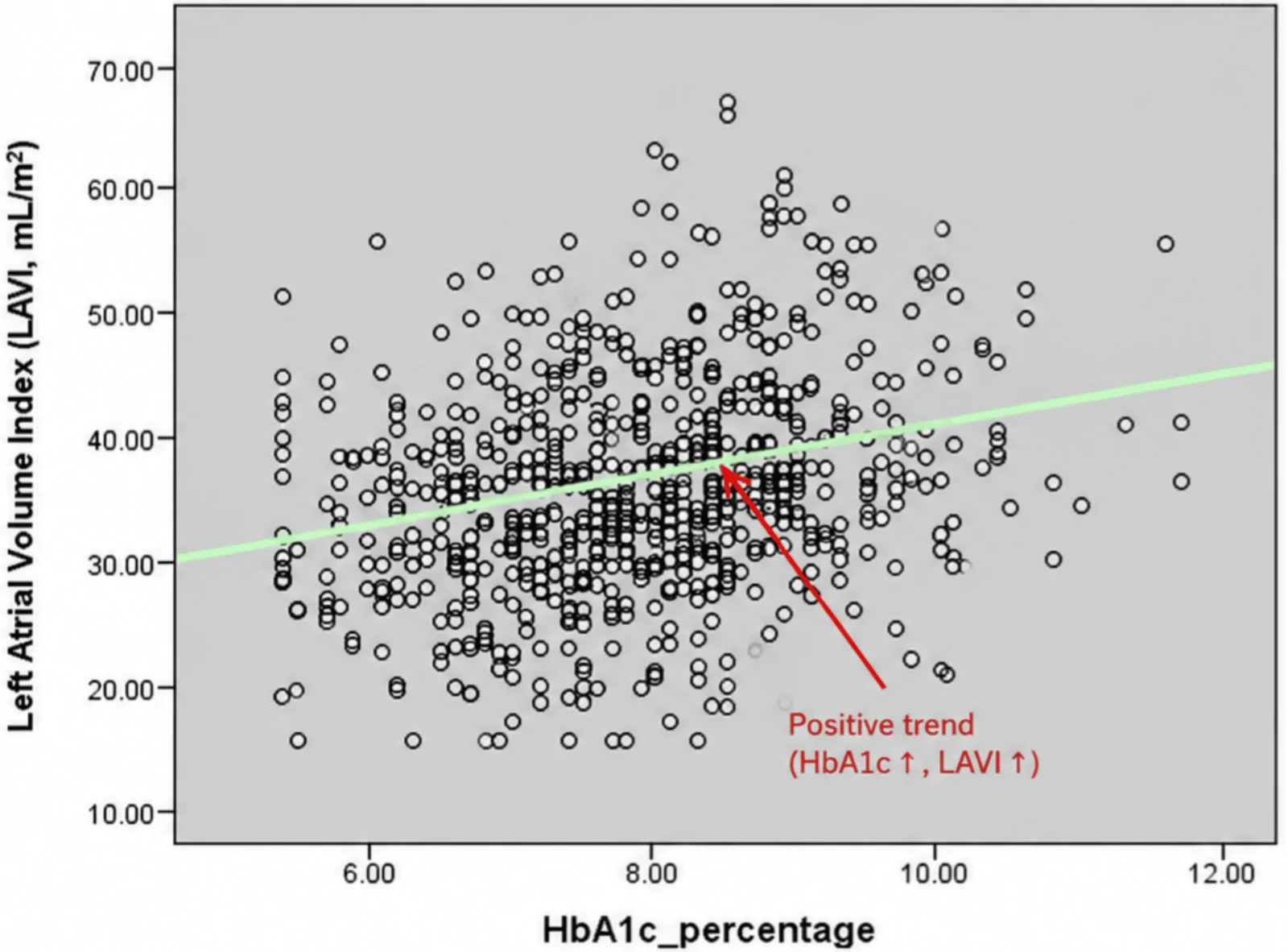
Scatterplot of glycated hemoglobin (HbA1c) and left atrial volume index (LAVI). Spearman’s rank correlation analysis was performed in 700 patients and demonstrated a significant positive correlation between HbA1c and LAVI (ρ = 0.287, p < 0.001).

Table 3 summarizes the multivariable linear regression analysis of factors independently associated with LAVI. The overall model was statistically significant, F(13, 686) = 48.860, p < 0.001, explaining 48.1% of the variance in LAVI (R² = 0.481; adjusted R² = 0.471). Higher age, BMI, and HbA1c were independently associated with higher LAVI. Among comorbidities, hypertension, CAD, heart failure, and chronic kidney disease were significantly associated with higher LAVI, while permanent AF was associated with higher LAVI compared with paroxysmal AF. In contrast, higher LVEF was independently associated with lower LAVI. Duration of diabetes, persistent AF, insulin treatment, and combined insulin/oral treatment were not significantly associated with LAVI. VIF values ranged from 1.087 to 2.357, indicating no evidence of problematic multicollinearity, and Cook’s distance indicated no influential observations.

**Table 3:** Multiple linear regression predicting LAVI (N = 700). Dependent variable: LAVI (mL/m²). The overall regression model was statistically significant, F(13, 686) = 48.860, p < 0.001, explaining 48.1% of the variance in LAVI (R = 0.693, R² = 0.481, adjusted R² = 0.471). No influential observations were identified, with Cook’s distance ranging from 0.000 to 0.020 (mean = 0.001), indicating acceptable influence diagnostics. B: unstandardized regression coefficient; SE: standard error; β: standardized regression coefficient; CI: confidence interval; VIF: variance inflation factor; LAVI: left atrial volume index; LL: lower limit; UL: upper limit; BMI: body mass index; AF: atrial fibrillation

Predictor	B	SE	Standardized β	t	95% CI for B (LL, UL)	p	VIF
Constant	10.776	4.259	-	2.531	(2.415, 19.138)	0.012	-
Age (years)	0.173	0.033	0.178	5.275	(0.109, 0.237)	<0.001	1.501
BMI (kg/m²)	0.306	0.062	0.142	4.952	(0.185, 0.427)	<0.001	1.087
Duration of diabetes (years)	0.069	0.051	0.047	1.347	(−0.032, 0.170)	0.178	1.598
HbA1c (%)	1.678	0.243	0.236	6.894	(1.200, 2.156)	<0.001	1.552
Left ventricular ejection fraction (LVEF, %)	−0.187	0.048	−0.133	−3.868	(−0.282, −0.092)	<0.001	1.557
Hypertension (yes vs. no)	3.044	0.510	0.180	5.972	(2.043, 4.045)	<0.001	1.197
Coronary artery disease (yes vs. no)	1.445	0.557	0.077	2.593	(0.351, 2.539)	0.010	1.161
Heart failure (yes vs. no)	5.091	0.709	0.234	7.181	(3.699, 6.484)	<0.001	1.401
Chronic kidney disease (yes vs. no)	2.554	0.667	0.111	3.827	(1.244, 3.864)	<0.001	1.111
AF persistent vs. paroxysmal	0.535	0.613	0.031	0.873	(−0.669, 1.739)	0.383	1.616
AF permanent vs. paroxysmal	1.559	0.688	0.088	2.266	(0.208, 2.910)	0.024	2.007
Insulin treatment vs. oral treatment	1.250	0.869	0.061	1.437	(−0.458, 2.957)	0.151	2.357
Both treatments vs. oral treatment	0.105	0.606	0.006	0.174	(−1.084, 1.295)	0.862	1.567

Table 4 shows that the positive association between HbA1c and LAVI remained statistically significant after additional adjustment for diastolic dysfunction. In the primary model, each 1% increase in HbA1c was associated with a 1.678 mL/m² increase in LAVI (B = 1.678, standard error (SE) = 0.243, β = 0.236, 95% confidence interval (CI) (1.200, 2.156), p < 0.001). After additional adjustment for diastolic dysfunction, the association remained significant and the HbA1c coefficient increased slightly to 1.781 mL/m² per 1% increase (B = 1.781, SE = 0.192, β = 0.251, 95% CI (1.404, 2.159), p < 0.001), representing a 6.1% increase in the coefficient relative to the primary model. Overall, the persistence of the significant association after adjustment indicates that the positive relationship between HbA1c and LAVI was robust to additional adjustment for diastolic dysfunction.

**Table 4:** Sensitivity analysis of the association between HbA1c and LAVI after additional adjustment for DD (N = 700). Percentage change was calculated relative to the HbA1c coefficient in the primary model. The primary model included age, BMI, diabetes duration, hypertension, coronary artery disease, heart failure, chronic kidney disease, left ventricular ejection fraction, atrial fibrillation type (dummy-coded), and diabetes treatment (dummy-coded). The sensitivity model additionally adjusted for DD grade. B: unstandardized regression coefficient; SE: standard error; β: standardized regression coefficient; CI: confidence interval; LL: lower limit; UL: upper limit; DD: diastolic dysfunction; LAVI: left atrial volume index; BMI: body mass index

Model	HbA1c B	SE	Standardized β	95% CI for B (LL, UL)	p	Change in B (%)
Primary model	1.678	0.243	0.236	(1.200, 2.156)	<0.001	-
Model additionally adjusted for DD	1.781	0.192	0.251	(1.404, 2.159)	<0.001	+6.1

Table 5 shows the difference in LAVI between those with good and poor glycemic control. Participants with poor glycemic control (HbA1c level ≥ 7%) had significantly higher LAVI ranks compared to participants with good glycemic control (HbA1c level < 7%) (mean rank: 372.58 vs. 274.14). The comparison revealed a statistically significant difference between the groups (U = 30,636.50, Z = −5.37, p < 0.001), with a small effect size (r = 0.20). These results suggest that the subjects with worse glycemic control tended to have higher LAVI values.

**Table 5:** Mann-Whitney U test comparing LAVI by glycemic control group (N = 700). LAVI was measured in mL/m². r represents the effect size for the Mann-Whitney U test and was calculated as r = |Z|/√N, where Z is the standardized test statistic and N is the total sample size. For this analysis, r = 5.37/√700 = 0.20. ***p < 0.001 was considered significant. LAVI: left atrial volume index; HbA1c: glycated hemoglobin; IQR: interquartile range

Variable	HbA1c group	N	LAVI, median (IQR) (mL/m²)	Mean rank	Sum of ranks	U	Z	p	Effect size (r)
LAVI (mL/m²)	Good control, HbA1c < 7%	157	34.80 (10.20)	274.14	43,039.50	30,636.50	−5.37	<0.001***	0.2
	Poor control, HbA1c ≥ 7%	543	38.50 (11.00)	372.58	202,310.50	-	-	-	-

Table 6 indicates that age, BMI, HbA1c, hypertension, heart failure, and chronic kidney disease were independently associated with increased odds of an enlarged LAVI. Specifically, each additional year of age was associated with a 4.6% increase in the odds of enlarged LAVI (odds ratio (OR) = 1.046, 95% CI 1.019-1.073, p = 0.001), while each 1 kg/m² increase in BMI was associated with a 9.2% increase in odds (OR = 1.092, 95% CI 1.039-1.148, p = 0.001). HbA1c showed a particularly strong association, with each 1% increase associated with 70.1% higher odds of enlarged LAVI (OR = 1.701, 95% CI 1.391-2.081, p < 0.001). Hypertension was associated with approximately 2.4-fold higher odds (OR = 2.389, 95% CI 1.612-3.540, p < 0.001), heart failure with approximately 4.1-fold higher odds (OR = 4.078, 95% CI 1.991-8.353, p < 0.001), and chronic kidney disease with approximately 2.6-fold higher odds (OR = 2.572, 95% CI 1.340-4.936, p = 0.005) of enlarged LAVI. In contrast, diabetes duration, LVEF, CAD, diabetes treatment, and AF type were not independently associated with enlarged LAVI (p > 0.05). The model demonstrated good discrimination, with an area under the curve (AUC) of 0.810 (95% CI 0.778-0.842, p < 0.001), while the Hosmer-Lemeshow test indicated adequate model fit (χ²(8) = 6.460, p = 0.596).

**Table 6:** Binary logistic regression analysis of factors associated with enlarged LAVI (N = 700). Model statistics (−2 log likelihood = 688.143; Cox and Snell R² = 0.254; Nagelkerke R² = 0.353; Hosmer-Lemeshow χ²(8) = 6.460; Hosmer-Lemeshow p = 0.596). The dependent variable was enlarged LAVI, coded 0 = normal LAVI (≤34 mL/m²) and 1 = enlarged LAVI (>34 mL/m²). The logistic regression model demonstrated an AUC of 0.810 (95% CI 0.778-0.842; p < 0.001). Adjusted OR represents Exp(B) after controlling for all other variables in the model. The reference category for each binary clinical variable was No. OR: odds ratio; CI: confidence interval; BMI: body mass index; HbA1c: glycated hemoglobin; LAVI: left atrial volume index; LVEF: left ventricular ejection fraction; SE: standard error; LL: lower limit; UL: upper limit; AF: atrial fibrillation; AUC: area under the curve

Predictor	B	SE	Wald χ²	df	OR	95% CI for OR (LL, UL)	p
Age (years)	0.044	0.013	11.338	1	1.046	(1.019, 1.073)	0.001
BMI (kg/m²)	0.088	0.025	12.035	1	1.092	(1.039, 1.148)	0.001
Diabetes duration	0.001	0.021	0.001	1	1.001	(0.960, 1.043)	0.972
HbA1c (%)	0.531	0.103	26.732	1	1.701	(1.391, 2.081)	<0.001
Diabetes treatment (overall)	-	-	0.610	2	-	-	0.737
Treatment category 1 vs. reference	0.267	0.360	0.553	1	1.307	(0.646, 2.644)	0.457
Treatment category 2 vs. reference	0.044	0.235	0.036	1	1.045	(0.660, 1.657)	0.850
AF type (overall)	-	-	1.489	2	-	-	0.475
AF category 1 vs. reference	0.124	0.233	0.284	1	1.132	(0.718, 1.786)	0.594
AF category 2 vs. reference	0.326	0.270	1.466	1	1.386	(0.817, 2.351)	0.226
LVEF (%)	−0.033	0.019	2.943	1	0.967	(0.931, 1.005)	0.086
Hypertension	0.871	0.201	18.837	1	2.389	(1.612, 3.540)	<0.001
Coronary artery disease (CAD)	0.337	0.237	2.022	1	1.401	(0.880, 2.229)	0.155
Heart failure	1.406	0.366	14.760	1	4.078	(1.991, 8.353)	<0.001
Chronic kidney disease	0.945	0.333	8.066	1	2.572	(1.340, 4.936)	0.005
Constant	−7.738	1.788	18.724	1	<0.001	-	<0.001

Table 7 demonstrates that the findings of the final logistic regression model were generally robust after internal validation using 1,000 bootstrap resamples. Age, BMI, HbA1c, hypertension, heart failure, and chronic kidney disease remained significant predictors of enlarged LAVI, with bootstrap estimates and CIs broadly consistent with the original model. HbA1c remained positively associated with enlarged LAVI (B = 0.531, OR = 1.701, bootstrap 95% CI for OR 1.395-2.181, p = 0.001), indicating that higher glycemic burden was associated with greater odds of enlarged LAVI. Age (OR = 1.046, bootstrap 95% CI 1.022-1.074, p = 0.001) and BMI (OR = 1.092, bootstrap 95% CI 1.039-1.155, p = 0.002) also remained significant. Hypertension (OR = 2.389, bootstrap 95% CI 1.623-3.770, p = 0.001), heart failure (OR = 4.080, bootstrap 95% CI 2.175-9.885, p = 0.001), and chronic kidney disease (OR = 2.573, bootstrap 95% CI 1.441-5.960, p = 0.005) continued to show increased odds of enlarged LAVI. In contrast, diabetes duration, diabetes treatment, AF type, LVEF, and CAD remained non-significant in the bootstrap analysis, with their bootstrap CIs including the null value. Overall, the persistence of the principal associations across 1,000 bootstrap resamples supports the stability and internal robustness of the final logistic regression model, particularly for the association between HbA1c and enlarged LAVI.

**Table 7:** Internal validation of the final logistic regression model using 1,000 bootstrap resamples. Internal validation was performed using 1,000 bootstrap resamples. Bootstrap CIs for ORs were obtained by exponentiating the bootstrap confidence limits for B. B: logistic regression coefficient; SE: standard error; OR: odds ratio; CI: confidence interval; BMI: body mass index; LVEF: left ventricular ejection fraction

Predictor	B	Bootstrap bias	Bootstrap SE	Bootstrap 95% CI for B	OR	Bootstrap 95% CI for OR	p
Age (years)	0.044	0.001	0.013	(0.022, 0.071)	1.046	(1.022, 1.074)	0.001
BMI (kg/m²)	0.088	0.001	0.027	(0.038, 0.144)	1.092	(1.039, 1.155)	0.002
Diabetes duration	0.001	0.000	0.023	(−0.045, 0.047)	1.001	(0.956, 1.048)	0.965
HbA1c (%)	0.531	0.015	0.111	(0.333, 0.780)	1.701	(1.395, 2.181)	0.001
Diabetes treatment (1)	0.267	0.009	0.386	(−0.512, 1.045)	1.306	(0.599, 2.843)	0.471
Diabetes treatment (2)	0.044	0.001	0.257	(−0.477, 0.543)	1.045	(0.621, 1.721)	0.868
Atrial fibrillation type (1)	0.124	0.013	0.246	(−0.354, 0.622)	1.132	(0.702, 1.863)	0.619
Atrial fibrillation type (2)	0.326	0.022	0.279	(−0.175, 0.903)	1.386	(0.839, 2.467)	0.231
LVEF (%)	−0.033	−0.001	0.019	(−0.072, 0.004)	0.968	(0.931, 1.004)	0.075
Hypertension	0.871	0.017	0.214	(0.484, 1.327)	2.389	(1.623, 3.770)	0.001
Coronary artery disease	0.337	0.008	0.242	(−0.137, 0.831)	1.401	(0.872, 2.296)	0.159
Heart failure	1.406	0.075	0.387	(0.777, 2.291)	4.080	(2.175, 9.885)	0.001
Chronic kidney disease	0.945	0.040	0.361	(0.365, 1.785)	2.573	(1.441, 5.960)	0.005

## Discussion

This study showed that glycemic control was positively associated with LA enlargement among patients with both AF and T2DM. Higher HbA1c levels were significantly associated with greater LAVI after adjustment for age, BMI, duration of diabetes, hypertension, CAD, heart failure, chronic kidney disease, LVEF, AF type, and diabetes treatment. Specifically, each 1% increase in HbA1c was associated with a 1.678 mL/m² increase in LAVI in the primary multivariable model. In a sensitivity analysis adjusting for diastolic dysfunction, the association remained significant, although the coefficient increased modestly to 1.781 mL/m² per 1% increase in HbA1c. Wang et al. reported that poor glycemic control was associated with adverse atrial structural remodeling, including increased LAVI and atrial fibrosis [8]. In their study of patients undergoing coronary artery bypass graft surgery, patients with diabetes had significantly greater LAVI and atrial fibrosis than controls, and higher HbA1c levels were associated with markers of atrial structural remodeling [8]. These findings are consistent with our results, although our study specifically evaluated patients with concomitant AF and T2DM and demonstrated an independent association between HbA1c and LAVI after adjustment for clinically relevant covariates. Matsuda et al. demonstrated an association between poor glycemic control and atrial low-voltage areas in patients with AF [16]. Our results also add to that evidence by showing an independent relationship between HbA1c and LAVI after adjustment for clinically relevant clinical and echocardiographic covariates. In addition, patients with poor glycemic control had significantly higher LAVI values, and for every 1% increase in HbA1c, a patient's odds of having enlarged LAVI increased by 70.1%. Zapolski and Wysokiński [17] demonstrated an independent relationship between HbA1c and LAVI in patients with T2DM. Similarly, Garg et al. [18], using data from the Atherosclerosis Risk in Communities study, reported that diabetes and higher HbA1c were associated with impaired LA conduit function. While Garg et al. evaluated LA function using strain-based measures in a community-based population, our study assessed LAVI as a structural marker in patients with established AF and T2DM. Thus, their findings complement our results by suggesting that abnormal glycemic status may be associated with both structural and functional LA abnormalities [18].

Wang et al. reported that poor glycemic control was associated with atrial structural remodeling in patients with T2DM [8]. Chronic hyperglycemia and glucose dysregulation can stimulate oxidative stress, inflammation, myocardial fibrosis, and metabolic remodeling, leading to atrial structural and electrophysiological changes. Karakasis et al. [2] and Chen et al. [19] described the potential role of glucose metabolic dysregulation and related mechanisms in AF pathogenesis and progression. Complementary evidence also suggests that diabetes may affect the cardiac conduction system. Movahed et al. [20] reported that diabetes mellitus was independently associated with third-degree atrioventricular block in a large nationwide database study. Although their study evaluated cardiac conduction disease rather than atrial structural remodeling, these findings provide complementary evidence that diabetes may affect cardiac electrical function more broadly. Garg et al. and Gong et al. reported adverse changes in LA structure and function among patients with T2DM, with metabolic and renal factors being associated with atrial remodeling [18,21].

Importantly, HbA1c remained independently associated after adjusting for several established cardiovascular risk factors, suggesting that chronic glycemic burden may affect LA remodeling beyond the effects of conventional cardiometabolic comorbidities. In the current study, chronic kidney disease, heart failure, CAD, hypertension, and age were also independently associated with higher LAVI. These findings align with previous evidence showing increased LA volume and impaired atrial function in patients with T2DM, an independent association between longer diabetes duration and LAVI, and adverse LA structural and functional changes in the presence of hypertension and renal dysfunction [22-25]. The present study, however, suggests that the independent relationship between HbA1c and LAVI, and the combined effect of multiple cardiovascular comorbidities, extend the current evidence and warrant further study.

Increasing severity of diastolic dysfunction showed a particularly clear relationship with atrial enlargement, with progressively higher LAVI across Grades I, II, and III dysfunction. El Aouar et al. [26] demonstrated a relationship between increased LAVI and the severity of diastolic dysfunction, while Lim et al. [27] showed that changes in LA function and volume were associated with subsequent heart failure risk. In the primary multivariable logistic regression model, hypertension, heart failure, and chronic kidney disease were independently associated with enlarged LAVI. The sensitivity analysis demonstrated that the association between HbA1c and LAVI remained significant after additional adjustment for diastolic dysfunction. These findings suggest that LA remodeling is a complex process that may rely on metabolic, hemodynamic, and cardiovascular mechanisms that can operate in synergy.

Clinically, these findings suggest that HbA1c may be an easily accessible laboratory marker to identify individuals with AF and T2DM at increased risk for adverse atrial remodeling. However, these findings should not be viewed as proof that hyperglycemia directly causes LA enlargement. Instead, they suggest that there is an independent relationship between poor glycemic control and higher LAVI in this high-risk population. Further prospective studies are needed to determine whether glycemic control can prevent and/or reverse atrial remodeling and whether this leads to better AF and cardiovascular outcomes.

Limitations

This study has several limitations. First, it is a retrospective cross-sectional design, which renders it impossible to ascertain a temporal or causal relationship between HbA1c and LAVI. In addition, we obtained HbA1c and echocardiographic measurements from a single clinical assessment episode for each participant; therefore, we could not evaluate longitudinal changes in glycemic control and LA remodeling. We did not formally assess intraobserver and interobserver reproducibility of LAVI measurements because the study relied on retrospectively recorded echocardiographic measurements; therefore, we cannot completely exclude measurement variability. In addition, because echocardiographic measurements were obtained from routine clinical examinations, variation in image acquisition and measurement across examinations could not be completely excluded. Second, the study took place in a single tertiary care hospital, which could affect the generalizability of the results to other populations and healthcare settings. In addition, some covariates, especially heart failure and diastolic dysfunction, could be part of the pathway between metabolic dysregulation and LA remodeling. This may have led to overadjustment and underestimation of the observed relationship between HbA1c and LAVI in the multivariable models. Third, the reliance on existing medical records limited the availability of some potentially relevant factors, such as AF duration and burden, rhythm at the time of echocardiography, specific antidiabetic medications, rate- and rhythm-control therapies, glucose variability, lifestyle factors, and long-term blood pressure control. Additionally, LAVI was derived from routine echocardiographic data, and we did not evaluate more advanced assessments of atrial remodeling, including LA strain or cardiac magnetic resonance measures of fibrosis. Furthermore, because the analysis was restricted to records with complete clinical, laboratory, and echocardiographic information, we cannot exclude selection bias. A complete comparison between included and excluded records was not possible because comparable individual-level data were not available for all excluded records, and excluding 300 of the 1,000 screened records may have resulted in a final analytic cohort that differed systematically from the broader screened population. Finally, although we adjusted for several important clinical and echocardiographic variables, we cannot exclude residual confounding from unmeasured factors.

Future directions

Future research should use a prospective, multicenter study design with serial assessment of HbA1c and LAVI to better delineate the time course between glycation and atrial remodeling. Further studies should assess other metabolic risk factors, as well as the effects of specific glucose-lowering therapies, AF duration and burden, and rhythm-control strategies. Improved techniques such as LA strain and cardiac magnetic resonance assessment of LA fibrosis could help clarify this association. Finally, interventional studies are needed to assess whether improved glycemic control reduces LA remodeling and improves AF-related and cardiovascular outcomes.

## Conclusions

Finally, elevated HbA1c was independently associated with higher LAVI and greater odds of LA enlargement in patients with AF and T2DM. Hypertension, heart failure, and chronic kidney disease were also independently associated with enlarged LAVI. The association between HbA1c and LAVI remained significant after additional adjustment for diastolic dysfunction in sensitivity analysis, with a modest increase in the HbA1c coefficient. These findings support an association between glycemic control and LA remodeling in this high-risk population but cannot establish causality.
